# Structural Features and Anti-Inflammatory Activity of a Low-Molecular-Weight Oligosaccharide Fraction from Lotus Bee Pollen

**DOI:** 10.3390/foods15142512

**Published:** 2026-07-16

**Authors:** Gongliang Liu, Jinxia Guo, Lantao Li, Weidong Bai, Hong Wang

**Affiliations:** 1 Guangdong Provincial Key Laboratory of Lingnan Specialty Food Science and Technology, Key Laboratory of Green Processing and Intelligent Manufacturing of Lingnan Specialty Food, Ministry of Agriculture and Rural Affairs, College of Light Industry and Food Technology, Zhongkai University of Agriculture and Engineering, Guangzhou 510225, China; gongliangliu@126.com (G.L.);; 2 Academy of Contemporary Agricultural Engineering Innovations, Zhongkai University of Agriculture and Engineering, Guangzhou 510225, China

**Keywords:** lotus bee pollen, low-molecular-weight oligosaccharide, microwave-assisted extraction, structural characterization, anti-inflammatory activity, RAW264.7 macrophages

## Abstract

A novel low-molecular-weight water-soluble oligosaccharide fraction (LBPP-1) was prepared from lotus bee pollen via microwave-assisted extraction, followed by Sevag deproteinization and diethylaminoethyl (DEAE)-cellulose-52 chromatography purification. Ion chromatography (IC) indicated a glucose-rich composition (84.6 mol% glucose), with minor amounts of arabinose, glucosamine, and galactose. Fourier transform-infrared spectroscopy (FT-IR), methylation analysis, and ^1^H nuclear magnetic resonance (NMR) collectively supported the assignment of LBPP-1 as a glucan-rich, structurally heterogeneous oligosaccharide fraction containing candidate →4)-Glcp-rich domains and minor arabinose/galactose-related linkages. However, the marked difference between the IC composition and the sugar-type distribution estimated from PMAA peak areas limits quantitative interpretation of the residue proportions and branching architecture. Furthermore, in vitro biological assays demonstrated that LBPP-1 significantly attenuated lipopolysaccharide (LPS)-induced inflammatory responses in RAW264.7 macrophages. It effectively reduced the secretion of nitric oxide (NO) and the levels of inducible nitric oxide synthase (iNOS), interleukin (IL)-6, and tumor necrosis factor (TNF)-α in RAW264.7 macrophages. Reverse transcription–quantitative polymerase chain reaction (RT-qPCR) analysis further revealed that LBPP-1 selectively suppressed the mRNA expression of cyclooxygenase-2 (COX-2), IL-1β, IL-6, iNOS, and transforming growth factor (TGF)-β1. These findings collectively suggest that LBPP-1, as a bioactive carbohydrate fraction derived from lotus bee pollen, holds promise as a natural functional food ingredient for managing inflammation-related conditions.

## 1. Introduction

Bee pollen, a natural mixture of plant pollen, nectar and bee secretions, has been documented as a functional food since antiquity, with historical records in the Bible and ancient Egyptian texts [1]. Recognized as an “all-round nutritional food”, bee pollen provides all essential amino acids for humans and bioactive components including polysaccharides, proteins, lipids, and polyphenols [2]. Due to its abundant bioactive components, cost-effectiveness, and broad-spectrum biological activities, bee pollen has attracted increasing interest as a promising ingredient for functional foods and nutraceuticals.

Among its various constituents, polysaccharides have emerged as key functional components responsible for health-promoting effects. These complex carbohydrates, typically ranging from oligosaccharides to high-molecular-weight polymers, exhibit diverse bioactivities including immunomodulation [3], antioxidant [4], anti-tumor [5], and anti-inflammatory effects [6]. Although some studies have reported that lower-molecular-weight polysaccharide or oligosaccharide fractions can exhibit improved absorption or cellular uptake and retain significant biological activity, the relationship between molecular weight (MW) and bioactivity is polysaccharide-specific and context-dependent. For instance, low-MW chitosan oligosaccharides (~3–5 kDa) showed markedly higher intestinal absorption compared with high-MW chitosan in both Caco-2 cells and in vivo studies [7]. Pharmacokinetic investigations on fucoidan fractions of different sizes also demonstrated that low-MW forms displayed distinct absorption and distribution patterns compared to larger counterparts [8]. On the other hand, some β-glucans require relatively higher molecular weights or triple-helix conformations to optimally activate immune receptors, indicating that structural and conformational features are equally critical determinants of function [9]. Together, these findings suggest that low-MW polysaccharides may offer advantages in certain contexts, but direct comparative studies remain limited.

Chronic inflammation underlies numerous pathological conditions, including cardiovascular disease, diabetes, neurodegenerative disorders, and cancer, representing a major global health challenge [10]. At the molecular level, lipopolysaccharide (LPS)-triggered inflammatory responses in macrophages are primarily orchestrated through the activation of multiple signaling cascades, which collectively drive the transcriptional upregulation of pro-inflammatory mediators including cyclooxygenase (COX)-2, inducible nitric oxide synthase (iNOS), tumor necrosis factor (TNF)-α, interleukin (IL)-1β, and IL-6 [11]. Natural polysaccharides have been widely reported to exert anti-inflammatory effects through multiple mechanisms, making them promising candidates for functional food applications that aim to mitigate inflammation-related risks [12].

*Nelumbo nucifera* (lotus) has been widely valued in traditional medicine systems across Asia for its diverse therapeutic properties [13]. Previous studies have characterized a high-MW pectic polysaccharide (WNPP-2-RG, 3.8 × 10^5^ Da) from lotus bee pollen and reported its immunological properties [14]. However, the structural features and anti-inflammatory potential of low-MW carbohydrate fractions from lotus bee pollen remain insufficiently investigated. Low-MW oligosaccharides may differ from high-MW polysaccharides in solubility, diffusion behavior, and interaction with immune cells, although such effects are highly structure-dependent. Therefore, this study aimed to prepare a low-MW water-soluble oligosaccharide fraction from lotus bee pollen using microwave-assisted extraction and chromatographic purification, characterize its structural features using chromatographic and spectroscopic methods, and evaluate its anti-inflammatory effects in LPS-stimulated RAW264.7 macrophages. The findings are expected to provide a basis for further understanding bee-pollen-derived oligosaccharides as potential functional food ingredients.

## 2. Materials and Methods

### 2.1. Materials and Reagents

The lotus bee pollen was obtained from Guangdong Guiling Apiculture Technology Co., Ltd. (Meizhou, China). Fetal bovine serum was purchased from Ecoscience Biotechnology Co., Ltd. (Shanghai, China). Coomassie brilliant blue R-250, bovine serum albumin, DEAE-52 cellulose and HBSS were purchased from Beijing Labgic Technology Co., Ltd. (Beijing, China). Methylene blue and dextran standard (1152 Da) were obtained from Shanghai Yuanye Biotechnology Co., Ltd. (Shanghai, China). Dextran standards (5, 11.6, 23.8, 48.6, 80.9, 148, 273, 409.8 and 667.8 kDa) and LPS (from *Escherichia coli* O111:B4) were purchased from Sigma-Aldrich Chemical Co. (St. Louis, MO, USA). DMEM medium was sourced from Thermo Fisher Scientific, Inc. (Shanghai, China). Iodomethane, dimethyl sulfoxide was procured from Adamas Reagent Co., Ltd. (Shanghai, China). RAW264.7 cells were obtained from National Collection of Authenticated Cell cultures (Shanghai, China). Trizol reagent and reverse transcription kit were purchased from Life Technologies Inc. (Waltham, MA, USA). TNF-α, iNOS, and IL-6 ELISA kits were purchased from Shanghai Jianglai Biotechnology Co., Ltd. (Shanghai, China). The real-time fluorescence quantitative kit was supplied from TransGen Biotech Co., Ltd. (Beijing, China). The nitric oxide (NO) kit was purchased from Beyotime Biotech Co., Ltd. (Shanghai, China). Monosaccharide standards and methylation kits were purchased from Borui Sugar Biotechnology Research Co., Ltd. (Yangzhou, China). All other chemical reagents were of analytical grade.

### 2.2. Extraction of Crude Polysaccharide

Appropriate amounts of lotus bee pollen were weighed, and distilled water was added at a solid-to-liquid ratio of 1:31 (*w*/*v*). The mixture was homogenized at 10,000 rpm for 30 s, then subjected to microwave-assisted extraction at 82 °C, 570 W for 41 min using a synergistic extraction reactor (Sino Tech Manufacture Co., Ltd., Nanjing, China). These extraction conditions were selected based on preliminary optimization experiments (single-factor experiments followed by response surface methodology) in our laboratory. The extract was concentrated under reduced pressure at 55 °C and centrifuged at 4750× *g* for 10 min. Four volumes of anhydrous ethanol were added to the supernatant and kept at 4 °C for 24 h. The resulting precipitate was collected by centrifugation (4750× *g*, 10 min), and the crude polysaccharide was obtained after vacuum freeze-drying [15]. The yield of crude polysaccharide was 1.30 ± 0.15% (*w*/*w*) based on the dry weight of lotus bee pollen.

### 2.3. Purification of LBPP-1

Proteins were removed from crude LBPP using the Sevag method [16]. The aqueous phase was collected, dialyzed against distilled water, and lyophilized. The deproteined sample was further purified using a DEAE-52 cellulose column (26 mm × 40 cm) chromatography, eluted sequentially with distilled water and NaCl solutions (0.1–0.5 M) at a flow rate of 2.0 mL/min. Fractions were collected and analyzed using the phenol–sulfuric acid method [17]. Based on the elution profile, three fractions were obtained: LBPP-1 (eluted with distilled water), LBPP-2 (eluted with 0.3 M NaCl), and LBPP-3 (eluted with 0.4 M NaCl). Although LBPP-2 was the major fraction obtained from the DEAE-52 column, LBPP-1 was selected for further characterization because it showed a more symmetrical single peak in HPGPC analysis, indicating better apparent homogeneity. Considering that reliable structural interpretation and bioactivity assessment require a relatively homogeneous carbohydrate fraction, LBPP-1 was used as the representative low-molecular-weight fraction in the present study.

### 2.4. Structural Characterization Analysis of LBPP-1

#### 2.4.1. High-Performance Gel Permeation Chromatography Analysis for Molecular Weight

The molecular weight (MW) of LBPP-1 was determined by high-performance gel permeation chromatography (HPGPC) using a BRT105-104-102 tandem gel column (8 × 300 mm). The column was set to 40 °C with ultrapure water as the mobile phase at a flow rate of 0.6 mL/min. A 20 μL sample (5 mg/mL) was injected, and MW was calculated using a calibration curve generated from dextran standards [15].

#### 2.4.2. Ion Chromatography Analysis for Monosaccharide Composition

Monosaccharide composition was analyzed by ion chromatography (IC) based on a modified method of Li et al. [10]. LBPP-1 (5 mg) was hydrolyzed with 2 mL of 3.0 M trifluoroacetic acid solution at 120 °C for 3 h. The hydrolysate was dried under nitrogen, re-dissolved in deionized water, and centrifuged (13,500× *g*, 5 min). Analysis was performed using a Dionex^TM^ ICS-5000 ion chromatography system (Thermo Fisher Scientific Inc., Waltham, MA, USA) equipped with a Carbopac^TM^ PA20 column (3 × 150 mm, Thermo Fisher Scientific Inc., Waltham, MA, USA) at 30 °C, with a 5 μL injection volume. The mobile phase consisted of (A) H_2_O, (B) 15 mM NaOH, and (C) 15 mM NaOH and 100 mM sodium acetate at 0.3 mL/min. The identification and quantification were performed using monosaccharide standards.

#### 2.4.3. UV-Vis Spectroscopy

The UV-Vis spectrum of LBPP-1 was recorded using a TU-9101 spectrophotometer (Beijing Purkinje General Instrument Co., Ltd., Beijing, China) across a wavelength range of 200–800 nm.

#### 2.4.4. Fourier-Transform Infrared (FT-IR) Spectroscopy

Approximately 1 mg of LBPP-1 was mixed with 100 mg dried KBr powder and pressed into pellets for analysis. FT-IR spectra were recorded using of a IRAffinity-1S spectrometer (Shimadzu, Shanghai, China) in the range of 4000–400 cm^−1^.

#### 2.4.5. Scanning Electron Microscopy (SEM)

The morphology of LBPP-1 observed using a SU8100 SEM (Hitachi, Tokyo, Japan). The powdered sample was mounted on a copper stub, sputter-coated with gold, and observed at an accelerating voltage of 2 kV.

#### 2.4.6. Methylation Analysis

Glycosidic linkages were identified using GC-MS after methylation based on a modified Shen method [18]. The fully methylated sample was subjected to hydrolysis, reduction, and acetylation, followed by GC-MS analysis.

#### 2.4.7. NMR Analysis

The sample was dissolved in 0.5 mL D_2_O to a final concentration of 40 mg/mL. ^1^H NMR was recorded at 25 °C using a Bruker AVANCE NEO 500 MHz spectrometer (Bruker, Rheinstetten, Germany). The spectrum was recorded in D_2_O without water presaturation and chemical shifts were referenced to the residual HDO signal. The strong residual HDO peak was taken into account when interpreting the anomeric region of the spectrum.

### 2.5. RAW264.7 Cells Culture

RAW264.7 macrophages were cultured in DMEM supplemented with 10% fetal bovine serum and 1% penicillin-streptomycin. Cells were maintained at 37 °C in a humidified incubator with 5% CO_2_.

### 2.6. Cell Viability Assay

The effect of LBPP-1 on the cell viability was determined using the methylene blue method [19]. RAW264.7 cells were seeded in 96-well plates (5 × 10^4^ cells/well) and incubated for 24 h. Cells were then treated with different concentrations of LBPP-1 (100–1000 µg/mL) for another 24 h. After removing the medium, 50 μL methylene blue solution (3.0 g of methylene blue and 12.5 mL of 50% glutaraldehyde were added in 500 mL of HBSS) was added for 1 h. After washing, 100 μL of eluent (4 mL of acetic acid and 250 mL of absolute ethanol were added in 245 mL of PBS) was added, and absorbance was measured at 595 nm. Cell viability (%) was calculated as:

Cell viability (%) = (A_1_ − A_0_)/(A_2_ − A_0_) × 100

where A_1_ is the absorbance of the LBPP-1-treated group (cells incubated with 100–1000 μg/mL LBPP-1 dissolved in PBS), A_2_ is the absorbance of the PBS control group (cells treated with an equal volume of PBS without LBPP-1), and A_0_ is the absorbance of the blank (wells containing culture medium without cells).

### 2.7. Effects on NO and Inflammatory Cytokines

RAW264.7 macrophages (5 × 10^5^ cells/well) were seeded in 6-well plates, and incubated for 24 h. Cells were divided into the following groups: (1) blank control group (untreated cells in DMEM); (2) LPS model control group (0.1 μg/mL LPS from *E. coli* O111:B4); (3–5) LBPP-1 treatment groups (300, 400, and 500 μg/mL LBPP-1 + LPS). After 24 h, culture supernatants were collected for NO, IL-6, and TNF-α measurements. Cells were then lysed with RIPA buffer, and the cell lysates were collected for iNOS measurement by ELISA. NO production was assessed using the Griess reagent, and levels of IL-6 and TNF-α were measured by ELISA kits.

### 2.8. mRNA Expression of Inflammatory Cytokines

Following the protocol of Bai et al. [20] with modification, RAW264.7 cells were cultured and grouped as described in Section 2.7. Total RNA was extracted using the Trizol reagent, and cDNA was synthesized using a reverse transcription kit. Quantitative real-time PCR (qRT-PCR) was performed using GAPDH as the internal control. The thermal cycling conditions were: 95 °C for 30 s (initial denaturation), followed by 40 cycles of 95 °C for 10 s and 60 °C for 30 s, with a final melting curve analysis. Primer sequences are listed in Table S1.

### 2.9. Statistical Analysis

All experiments were performed in triplicate. Data were expressed as mean ± standard deviation (SD). Statistical significance (*p* < 0.05) was determined by one-way ANOVA with Tukey’s test out using SPSS 26, and graphs were generated using Origin 2021.

## 3. Results and Discussion

### 3.1. Isolation and Purification of LBPP Fractions

**Figure 1:**
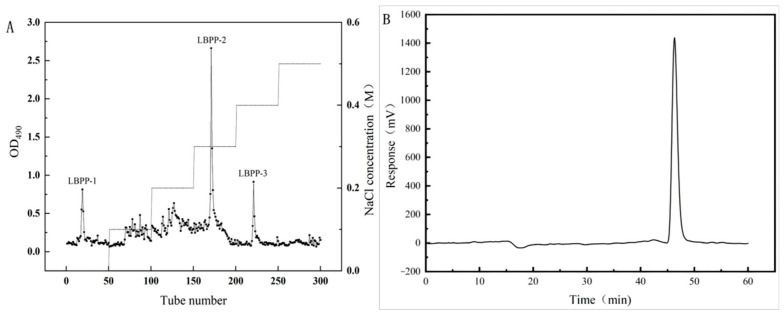
Isolation and molecular weight characterization of LBPP-1. (**A**) Elution profile of deproteinized lotus bee pollen crude polysaccharides on a DEAE-cellulose-52 ion-exchange column; absorbance at 490 nm was monitored using the phenol–sulfuric acid method. (**B**) HPGPC chromatogram of LBPP-1, showing a single symmetrical peak indicative of compositional homogeneity; molecular weight was determined by comparison with dextran standards.

Crude polysaccharides were extracted from lotus bee pollen with a yield of 1.30 ± 0.15% (*w*/*w*) based on dry pollen weight and subsequently deproteinized using the Sevag method prior to fractionation by DEAE-52 cellulose ion-exchange chromatography. The elution profile monitored by the phenol–sulfuric acid method revealed three distinct elution peaks, indicating effective separation of polysaccharide fractions with different charge densities (Figure 1A). The fractions eluted with deionized water, 0.3 M NaCl, and 0.4 M NaCl were designated as LBPP-1, LBPP-2, and LBPP-3, respectively. Among these fractions, LBPP-1 exhibited a single, symmetric elution peak in subsequent analyses and was therefore selected for detailed structural characterization and biological evaluation.

### 3.2. Structural Characterization of LBPP-1

#### 3.2.1. Molecular Weight Determination

High-performance gel permeation chromatography (HPGPC) analysis revealed that LBPP-1 exhibited a single symmetrical peak (Figure 1B), indicating apparent size homogeneity under the chromatographic conditions used. Based on dextran standard calibration, the molecular weight of LBPP-1 was calculated as 1625 Da, which was significantly lower than that of WNPP-2-RG (3.8 × 10^5^ Da), a high-molecular-weight pectic polysaccharide previously isolated from lotus bee pollen [14]. Because HPGPC separates mainly according to hydrodynamic size, a single peak does not exclude the presence of closely sized or co-eluting oligosaccharides. Direct mass spectrometry would be required to confirm the exact molecular ions and degree of polymerization. This substantial reduction in molecular weight is primarily attributed to microwave-assisted extraction (570 W, 41 min), which generates localized heating and mechanical shear, selectively cleaving weaker glycosidic linkages within the native polysaccharide chain. Microwave-assisted extraction has been widely demonstrated to reduce the molecular size of natural polysaccharides while maintaining their functional groups and bioactivity [21]. This extraction method preferentially breaks weaker glycosidic bonds while preserving the core structural integrity, resulting in the release of low-molecular-weight bioactive fragments [22]. Consequently, LBPP-1 should be regarded as an oligosaccharide fraction or a degraded polysaccharide fragment rather than a fully sequence-defined single molecule.

#### 3.2.2. Monosaccharide Composition

**Figure 2:**
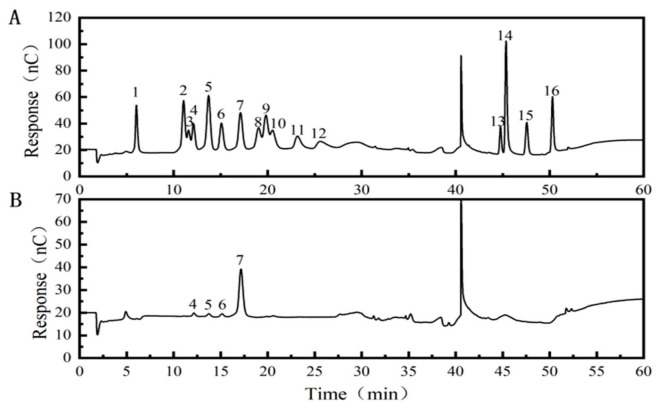
Monosaccharide composition analysis by IC. (**A**) Monosaccharide standard mixtures; (**B**) Monosaccharide composition of LBPP-1. 1 fucose, 2 galactosamine, 3 rhamnose, 4 arabinose, 5 glucosamine, 6 galactose, 7 glucose, 8 N-acetyl-glucosamine, 9 xylose, 10 mannose, 11 fructose, 12 ribose, 13 galactose acid, 14 guluronic acid, 15 glucuronic acid, 16 mannuronic acid.

Ion chromatography analysis revealed that LBPP-1 was an oligosaccharide composed of arabinose (Ara), glucosamine (GlcN), galactose (Gal), and glucose (Glc) in a molar ratio of 0.064:0.027:0.064:0.846 (Figure 2). The predominance of glucose (84.6 mol%) suggests that LBPP-1 is primarily glucan-based, consistent with the thermal hydrolysis and selective cleavage of glycosidic linkages facilitated by microwave treatment [23].

#### 3.2.3. UV-Vis Spectral Analysis

**Figure 3:**
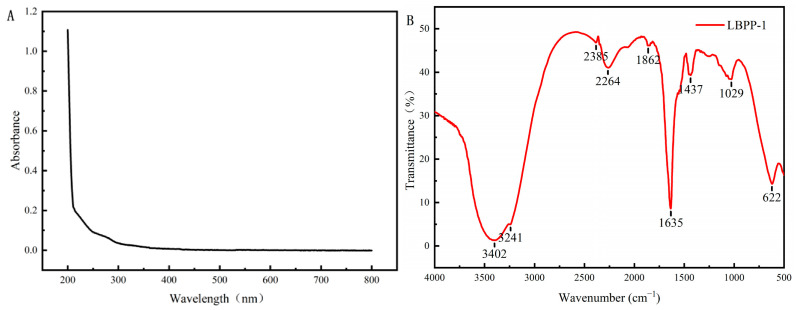
Spectral analysis of LBPP-1. (**A**) UV-Vis spectrum; (**B**) FT-IR spectrum.

The UV-Vis spectrum of LBPP-1 exhibited no detectable absorption at 260 nm or 280 nm (Figure 3A), indicating the absence of nucleic acids and proteins, which suggested the effective deproteinization and high purity of LBPP-1.

#### 3.2.4. FT-IR Spectroscopy

FT-IR spectrum of LBPP-1 (Figure 3B) revealed characteristic oligosaccharide absorption bands. The broad and intense absorption band at 3402 cm^−1^ was attributed to O-H stretching vibration of hydroxyl groups [24]. The absorption band at 1635 cm^−1^ is likely attributed to the bending vibration of bound water within the oligosaccharide, implying the existence of a hydrated form of the oligosaccharide [25]. The absorption at 1437 cm^−1^ was assigned to C–H bending vibrations of methylene groups, and the band at 1029 cm^−1^ was assigned to C-O-C stretching in the glucose residue, confirming the pyranose ring backbone of the oligosaccharide [26]. Diagnostic anomeric bands around 890 cm^−1^ (β-linkage) and 840 cm^−1^ (α-linkage) were not clearly resolved; therefore, FT-IR alone was not used to assign the anomeric configuration.

#### 3.2.5. Morphological Characteristics

**Figure 4:**
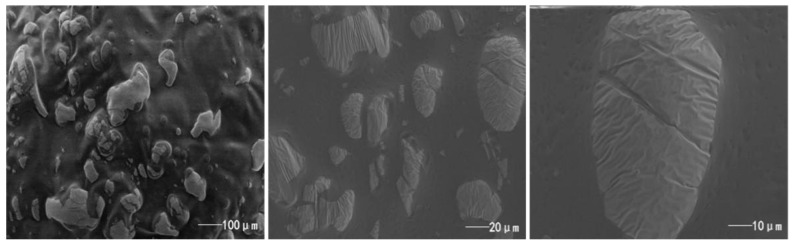
Scanning electron micrographs of LBPP-1 morphology at different magnifications.

SEM imaging revealed hierarchical structural features of oligosaccharide (Figure 4). At 500× magnification, LBPP-1 appeared as irregular ovoid particles with size heterogeneity. Higher magnification (2000×) showed granular surfaces with fragmentation, while 5000× imaging revealed striated fissures and porous architectures. The observed porous and gully-like microstructure is consistent with the structural disruption typically induced by rapid heating and pressure changes during microwave-assisted extraction [27].

#### 3.2.6. Glycosidic Linkage Analysis

To elucidate the glycosidic linkage patterns of the Ara, Glc and Gal residues within LBPP-1, methylation analysis was performed, followed by hydrolysis, reduction, acetylation, and analysis via GC-MS. This analysis yielded a total of fifteen distinct partially methylated alditol acetate (PMAA) derivatives (Table 1). These derivatives included: for arabinofuranose (Araf)-2,3,5-Me_3_-Araf, 3,5-Me_2_-Araf, 2,3-Me_2_-Araf and 3-Me_1_-Araf; for glucopyranose (Glcp) 2,3,4,6-Me_4_-Glcp, 2,4,6-Me_3_-Glcp, 2,3,6-Me_3_-Glcp, 2,3,4-Me_3_-Glcp, 2,6-Me_2_-Glcp, and 2,3-Me_2_-Glcp; for galactopyranose (Galp) 2,3,4,6-Me_4_-Galp, 2,3,6-Me_3_-Galp, 2,4,6-Me_3_-Galp, 2,3,4-Me_3_-Galp, and 2,3-Me_2_-Galp.

The identified linkage patterns aligned with established methodologies for interpreting methylation data [28]. Among the quantified PMAA derivatives, the →4)-linked glucopyranosyl residue (4-Glcp-) constituted the largest proportion. However, summing the methylation-derived PMAA peak areas by sugar type gave a higher relative arabinose contribution and a lower relative glucose contribution than the IC monosaccharide composition. Specifically, the proportion of arabinose-derived PMAAs (~35%) was higher than the arabinose content measured by IC (6.4 mol%), while glucose-derived PMAAs (~53%) were lower than the glucose content (84.6 mol%). This discrepancy may arise from incomplete methylation or hydrolysis of poorly soluble 4-linked glucan domains, different derivatization efficiencies, and unequal GC-MS response factors among PMAA derivatives. However, the magnitude of this discrepancy cannot be fully resolved by the available data. Therefore, the IC values are treated as method-specific estimates rather than absolute compositional data, while the methylation data are interpreted qualitatively for candidate linkage identification and are not presented as an independent quantitative composition measurement. The two datasets are not treated as quantitatively concordant, and the precise residue distribution and branching architecture remain unresolved.

Glucosamine (GlcN) was detected at 2.7 mol% by IC analysis, but no amino-sugar derivatives were identified in the methylation analysis (Table 1). This is likely because GlcN residues are typically present as N-acetylglucosamine (GlcNAc) in native glycans. During the acid hydrolysis step of methylation analysis, the N-acetyl group may be partially or completely cleaved, and the resulting free amino group may not be efficiently derivatized under the standard methylation conditions (which target hydroxyl groups), leading to its absence in the GC-MS profile. Furthermore, previous studies have shown that the PMAA of glucosamine can exhibit preferential loss on GC columns at low injection amounts and a molar response factor approximately 25% higher than that of galactose PMAA, further complicating its detection and quantification [29]. The presence of GlcN in the lotus bee pollen oligosaccharide fraction may originate from fungal cell wall components (chitin or chitosan) associated with pollen-colonizing microorganisms, or from natural glycoprotein constituents of pollen. Given its low abundance and uncertain structural assignment, GlcN was not included in the proposed linkage model.

The predominance of →4)-Glcp-(1→ linkage is reminiscent of cellulose; however, LBPP-1 is fully water-soluble, which is consistent with the short chain length and the presence of arabinose- and galactose-containing side chains that disrupt linear chain packing. Considering the low apparent molecular weight of LBPP-1 and the detection of multiple linkage environments, LBPP-1 is more conservatively interpreted as a mixture of closely sized glucan-rich hetero-oligosaccharides containing →4)-Glcp-rich domains and minor Ara/Gal-associated linkages.

#### 3.2.7. ^1^H NMR Analysis

**Figure 5:**
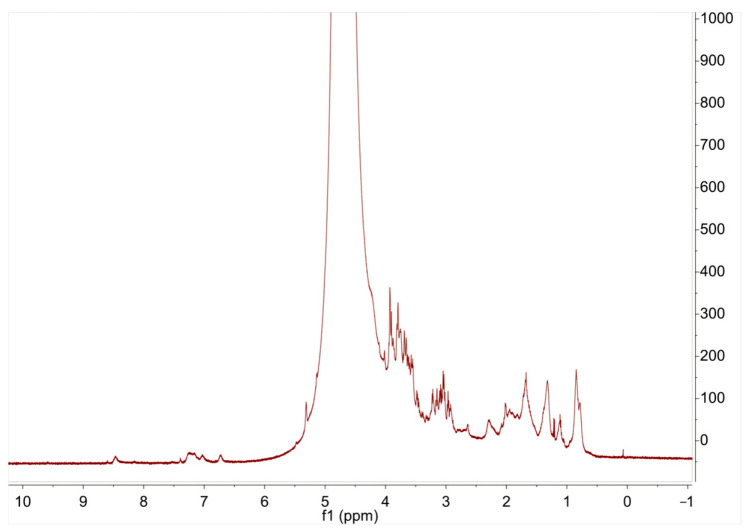
Expanded ^1^H NMR spectrum of LBPP-1. The spectrum is shown over a narrowed range so that carbohydrate proton signals are visible; the strong signal around δ 4.70–4.80 ppm corresponds mainly to residual HDO.

The ^1^H NMR spectrum of LBPP-1 (Figure 5) showed typical carbohydrate proton signals in the δ 3.0–5.5 ppm region [30]. The strong signal at approximately δ 4.70–4.80 ppm was assigned mainly to residual HDO and was not used as direct evidence for anomeric configuration. After narrowing the displayed spectral range, several carbohydrate-associated resonances became visible, including signals in the δ 3.0–4.0 ppm region attributable to ring protons and weak signals around δ 5.0–5.2 ppm that may be likely attributed to α-anomeric protons of arabinofuranosyl residues [31]. Taken together with the methylation results, the ^1^H NMR data support the carbohydrate nature and heterogeneity of LBPP-1, but they do not independently establish the complete sequence or α/β configuration. Further 2D NMR would be required for definitive structural elucidation.

### 3.3. Anti-Inflammatory Activity of LBPP-1 in LPS-Stimulated RAW264.7 Macrophages

To evaluate the anti-inflammatory potential of LBPP-1, a series of in vitro experiments were conducted using LPS-stimulated RAW264.7 cells, a widely used model for studying macrophage-mediated inflammation.

#### 3.3.1. Effect of LBPP-1 on RAW264.7 Cells Viability

Prior to assessing the anti-inflammatory activity, the cytotoxicity of LBPP-1 on RAW264.7 cells was evaluated using the methylene blue method. As shown in Figure S1, treatment with LBPP-1 (0–1000 μg/mL) for 24 h did not significantly reduce cell viability, indicating that the oligosaccharide is non-toxic to macrophages at the tested concentrations. In fact, at concentrations of 100 and 200 μg/mL, LBPP-1 exhibited a slight but statistically significant increase in cell proliferation (*p* < 0.05), with the viability reaching 123% compared with the untreated control. At concentrations above 400 μg/mL, the proliferative effect diminished, and cell viability returned to levels comparable to those of the untreated control group (approximately 100%), indicating the absence of cytotoxicity across the entire tested concentration range. The initial increase in cell proliferation at 100–200 μg/mL may reflect a mild mitogenic or metabolic stimulatory effect of LBPP-1 at lower doses. It is worth noting that plant- or bee-derived polysaccharide fractions may potentially contain endotoxin contaminants; however, the multistep purification procedure—including Sevag deproteinization, extensive dialysis, and DEAE-52 chromatography—is expected to effectively remove such low-molecular-weight impurities. Furthermore, the absence of cytotoxicity at all tested concentrations (Figure S1) indirectly supports the safety of the LBPP-1 preparation. Direct endotoxin quantification by Limulus amebocyte lysate (LAL) assay will be included in future studies with larger-scale preparations. Overall, these results indicated that LBPP-1 is safe for use in subsequent anti-inflammatory assays.

#### 3.3.2. Effects of LBPP-1 on Inflammatory Mediator Secretion in LPS-Stimulated RAW264.7 Macrophages

**Figure 6:**
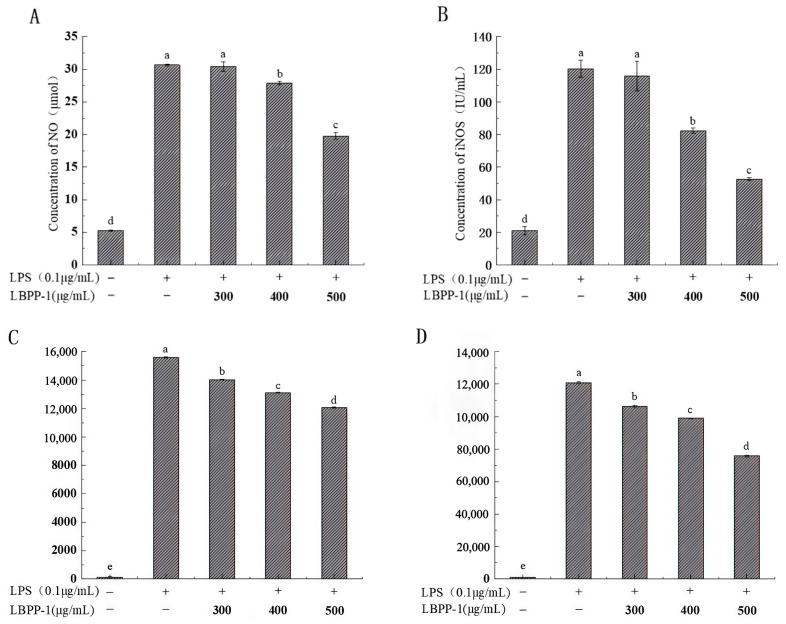
Effects of LBPP-1 on inflammatory mediator levels in LPS-stimulated RAW264.7 cells. (**A**) NO, (**B**) iNOS, (**C**) IL-6, (**D**) TNF-α. Different letters (a–e) indicate significant differences among groups (*p* < 0.05). The blank control group contained untreated cells, and the LPS group was used as the model control for evaluating LBPP-1 treatment effects.

The anti-inflammatory effects of LBPP-1 were further investigated by measuring the levels of NO, iNOS, IL-6, and TNF-α in the culture medium of LPS-induced RAW264.7 cells. LPS induction significantly elevated all measured inflammatory factors compared with the blank control group (*p* < 0.05), confirming successful establishment of the inflammation model (Figure 6). As shown in Figure 6A, compared with the LPS group, treatment with LBPP-1 at concentrations ranging from 300 to 500 μg/mL significantly reduced NO production. Specifically, at 500 μg/mL, the NO level was reduced to 19.77 μM, a 35% decrease compared to the LPS group. These results indicated that LBPP-1 effectively inhibited the nitrosative stress associated with inflammation. Furthermore, the level of iNOS in cell lysates was markedly suppressed by LBPP-1 (Figure 6B). The iNOS level in the LPS group was 120 IU/mL, but it was reduced to 52 IU/mL when treated with 500 μg/mL LBPP-1, representing a 57% reduction. Similarly, LBPP-1 significantly decreased the levels of IL-6 and TNF-α in a dose-dependent manner. At the concentrations of 400 μg/mL and 500 μg/mL, the levels of IL-6 and TNF-α were reduced by 16–23% and 18–37%, respectively (Figure 6C,D). The experimental results indicated that LBPP-1 can inhibit the levels of NO, iNOS, IL-6, and TNF-α in a dose-dependent manner, thereby attenuating LPS-induced inflammatory responses in RAW264.7 macrophages. These findings are consistent with those reported for other anti-inflammatory polysaccharides, such as lignified okra polysaccharides, which also ameliorate inflammation through inhibition of NO and pro-inflammatory cytokine [32].

#### 3.3.3. Effects of LBPP-1 on the mRNA Expression of Inflammatory Genes in LPS-Stimulated RAW264.7 Macrophages

**Figure 7:**
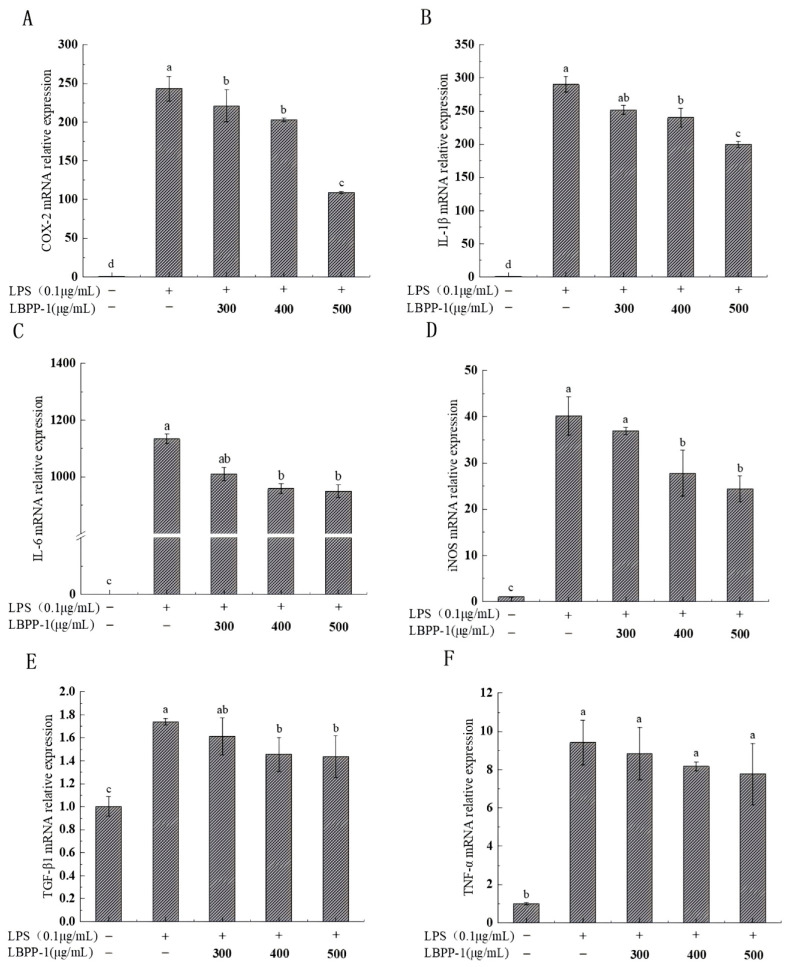
Effect of LBPP-1 on inflammatory gene expression at transcriptional levels in LPS-stimulated RAW264.7 cells. (**A**) COX-2, (**B**) IL-1β, (**C**) IL-6, (**D**) iNOS, (**E**) TGF-β1, (**F**) TNF-α. Different letters (a–d) indicate significant differences among groups (*p* < 0.05). The blank control group contained untreated cells, and the LPS group was used as the model control for evaluating LBPP-1 treatment effects.

To elucidate the molecular mechanism underlying the anti-inflammatory activity of LBPP-1, we examined its effect on inflammatory gene expression using qRT-PCR analysis of LPS-stimulated RAW264.7 macrophages. The results demonstrated that LBPP-1 treatment resulted in a dose-dependent inhibition of the mRNA expression of COX-2, IL-1β, IL-6, and iNOS and TGF-β1 in LPS-stimulated RAW264.7 cells, whereas TNF-α mRNA expression was not significantly affected at 300–500 μg/mL (Figure 7). At the concentration of 500 μg/mL, the mRNA expression of COX-2, IL-1β, IL-6, iNOS, and TGF-β1 was reduced by 55.1%, 17.5%, 39.2%, 16.3%, and 31.2% respectively, compared with the LPS group.

The dose-dependent effects observed across multiple genes suggested that LBPP-1 exerted concentration-dependent regulatory effects on inflammatory gene transcription. The varying degrees of suppression (from 16.3% for iNOS to 55.1% for COX-2) indicated differential sensitivity of inflammatory genes to the regulatory effect of LBPP-1, possibly reflecting distinct transcriptional control mechanisms or pathway preferences. The observed transcriptional inhibition of COX-2 and iNOS provided mechanistic insight into the ability of LBPP-1 to reduce NO production, as these enzymes were key regulators of prostaglandin and nitric oxide biosynthesis, respectively [32,33]. The significant suppression of IL-6 and IL-1β, which were central mediators in the cytokine cascade, further supported the potential of LBPP-1 to interrupt inflammatory amplification loops [34]. TGF-β1 was included as an inflammation-associated immunoregulatory factor rather than a classical pro-inflammatory cytokine, and its downregulation might be interpreted in the context of LPS-induced macrophage activation.

Notably, while LBPP-1 significantly reduced the protein secretion of TNF-α (Figure 6D), no significant reduction in its mRNA expression was observed at concentrations ranging from 300 to 500 μg/mL (*p* > 0.05). This discrepancy between mRNA and protein levels suggests that LBPP-1 might not suppress TNF-α strictly at the transcriptional level, a phenomenon also reported for certain *Sargassum fusiforme* polysaccharides [35]. Instead, it is highly probable that LBPP-1 exerts its inhibitory effects through post-transcriptional mechanisms, such as altering mRNA stability, inhibiting translation efficiency, or impeding the intracellular trafficking and secretion of the TNF-α protein. This differential and selective modulation indicates that LBPP-1 does not induce a global immunosuppressive shutdown, but rather fine-tunes the inflammatory cascade in a pathway-specific manner, which represents a favorable safety profile for functional food applications.

Furthermore, based on the structural characterization of LBPP-1, the oligosaccharide is predominantly composed of glucose (84.6 mol%) with a putative →4)-linked glucopyranosyl backbone. β-Glucans and glucan-rich oligosaccharides have been widely reported to exert immunomodulatory effects through interactions with pattern recognition receptors such as Dectin-1 on immune cells [9]. The presence of minor arabinose and galactose residues may contribute to the overall structural complexity and potentially influence receptor binding specificity [32], although the primary bioactive determinant is likely the glucan backbone. These findings position LBPP-1 as a promising anti-inflammatory agent with a unique mechanism of action that combines broad-spectrum cytokine modulation with selective pathway regulation.

## 4. Conclusions

In this study, a low-molecular-weight oligosaccharide fraction (LBPP-1; apparent MW ~1625 Da) was isolated from lotus bee pollen. The combined analytical data support the presence of candidate →4)-Glcp-rich domains and minor arabinose/galactose- containing branches. In addition, the marked difference between the IC composition and the sugar-type distribution estimated from PMAA peak areas prevents definitive quantification of residue proportions and branching architecture. In LPS-stimulated RAW264.7 macrophages, LBPP-1 was well tolerated and significantly attenuated inflammatory responses by reducing the production of NO, iNOS, IL-6, and TNF-α, as well as selectively downregulating the mRNA expression of key inflammatory genes. These results indicate that LBPP-1 exhibits notable in vitro anti-inflammatory activity and may serve as a promising lotus bee pollen–derived bioactive ingredient for functional food applications. Further studies involving advanced structural analysis (including MALDI-TOF or ESI-MS for definitive molecular weight confirmation, and 2D NMR for complete sequence elucidation), direct endotoxin quantification by LAL assay, positive anti-inflammatory controls, and pathway-level validation are needed to clarify its structure–function relationship and biological relevance.

## Figures and Tables

**Table 1:** GC-MS result of partially methylated alditol acetates (PMAAs) of methylation analysis *.

RT (min)	Methylated Sugar	Mass Fragments (*m*/*z*)	Molar Ratio	Type of Linkage
10.516	2,3,5-Me_3_-Araf	43, 71, 87, 101, 117, 129, 145, 161	0.199	t-Araf-(1→
13.997	3,5-Me_2_-Araf	43, 71, 87, 101, 129, 161, 189	0.024	→2)-Araf-(1→
15.477	2,3-Me_2_-Araf	43, 71, 87, 99, 101, 117, 129, 161, 189	0.083	→5)-Araf-(1→
16.982	2,3,4,6-Me_4_-Glcp	43, 71, 87, 101, 117, 129, 145, 161, 205	0.070	t-Glcp-(1→
17.908	2,3,4,6-Me_4_-Galp	43, 71, 87, 101, 117, 129, 145, 161, 205	0.022	t-Galp-(1→
19.113	3-Me_1_-Araf	43, 57, 85, 99, 117, 127, 129, 159, 189	0.040	→2,5)-Araf-(1→
20.914	2,4,6-Me_3_-Glcp	43, 87, 99, 101, 117, 129, 161, 173, 233	0.095	→3)-Glcp-(1→
21.080	2,3,6-Me_3_-Galp	43, 87, 99, 101, 113, 117, 129, 131, 161, 173, 233	0.023	→4)-Galp-(1→
21.451	2,3,6-Me_3_-Glcp	43, 87, 99, 101, 113, 117, 129, 131, 161, 173, 233	0.286	→4)-Glcp-(1→
21.835	2,4,6-Me_3_-Galp	43, 87, 99, 101, 117, 129, 161, 173, 233	0.027	→3)-Galp-(1→
22.189	2,3,4-Me_3_-Glcp	43, 87, 99, 101, 117, 129, 161, 189, 233	0.022	→6)-Glcp-(1→
23.748	2,3,4-Me_3_-Galp	43, 87, 99, 101, 117, 129, 161, 189, 233	0.015	→6)-Galp-(1→
24.453	2,6-Me_2_-Glcp	43, 87, 97, 117, 159, 185	0.020	→3,4)-Glcp-(1→
26.721	2,3-Me_2_-Glcp	43, 71, 85, 87, 99, 101, 117, 127, 159, 161, 201	0.033	→4,6)-Glcp-(1→
28.605	2,3-Me_2_-Galp	43, 71, 85, 87, 99, 101, 117, 127, 159, 161, 201, 261	0.038	→4,6)-Galp-(1→

* Araf, arabinofuranose; Glcp, glucopyranose; Galp, galactopyranose. Molar ratios were calculated from peak areas relative to the total of all identified PMAA derivatives.

## Data Availability

The original contributions presented in this study are included in the article/Supplementary Materials. Further inquiries can be directed to the corresponding author.

## Supplementary Materials

The following supporting information can be downloaded at: https://www.mdpi.com/article/10.3390/foods15142512/s1, Table S1: Target genes and primers sequences in qPCR; Figure S1: Effect of LBPP-1 on the viability of RAW264.7 cells.

## Abbreviations

The following abbreviations are used in this manuscript:

COX-2	cyclooxygenase-2
ELISA	enzyme-linked immunosorbent assay
FT–IR	fourier-transform infrared
GAPDH	glyceraldehyde-3-phosphate dehydrogenase
HBSS	Hanks’ balanced salt solution
HPGPC	high-performance gel permeation chromatography
IC	ion chromatography
IL-1β	interleukin-1β
IL-6	interleukin-6
iNOS	inducible nitric oxide synthase
LBPP	low-molecular-weight water-soluble oligosaccharide
LPS	lipopolysaccharide
MW	molecular weight
NMR	nuclear magnetic resonance spectroscopy
NO	nitric oxide
PBS	phosphate-buffered saline
qRT-PCR	quantitative real time-polymerase chain reaction
SD	standard deviation
SEM	scanning electron microscopy
TGF-β1	transforming growth factor-β1
TNF-α	tumor necrosis factor-α
UV-Vis	ultraviolet–visible spectroscopy
WNPP-2-RG	a high-molecular-weight pectic polysaccharide from lotus bee pollen
