# Supplementary material for: Structural Features and Anti-Inflammatory Activity of a Low-Molecular-Weight Oligosaccharide Fraction from Lotus Bee Pollen
Source: Foods. 2026 Jul 16;15(14):2512. doi: 10.3390/foods15142512 (PMC13409687; doi:10.3390/foods15142512)
Supplement: Supplementary file 1 [file foods-15-02512-s001.zip › foods-4408472-supplementary.pdf]

Table S1. Target genes and primers sequences in qPCR.

Gene name	GenBank ID	Amplicon size (bp)	Primer sequence
GAPDH	NM_001289726.2	123	F：AGGTCGGTGTGAACGGATTTG<br>R：TGTAGACCATGTAGTTGAGGTCA
COX-2	NM_011198.5	124	F：TGCACTATGGTTACAAAAGCTGG<br>R：TCAGGAAGCTCCTTATTTCCCTT
iNOS	NM_010927.4	127	F：GTTCTCAGCCCAACAATACAAGA<br>R：GTGGACGGGTCGATGTCAC
IL-1β	NM_008361.4	116	F：GAAATGCCACCTTTTGACAGTG<br>R：TGGATGCTCTCATCAGGACAG
TGF-β1	XM_036152883.1	142	F：CTTCAATACGTCAGACATTCGGG<br>R：GTAACGCCAGGAATTGTTGCTA
TNF-α	NM_013693.3	122	F：CTGAACTTCGGGGTGATCGG<br>R：GGCTTGTCACTCGAATTTTGAGA
IL-6	NM_001314054.1	205	F：CTGACAATATGAATGTTGGG<br>R：TCCAAGAAACCATCTGGCTAGG

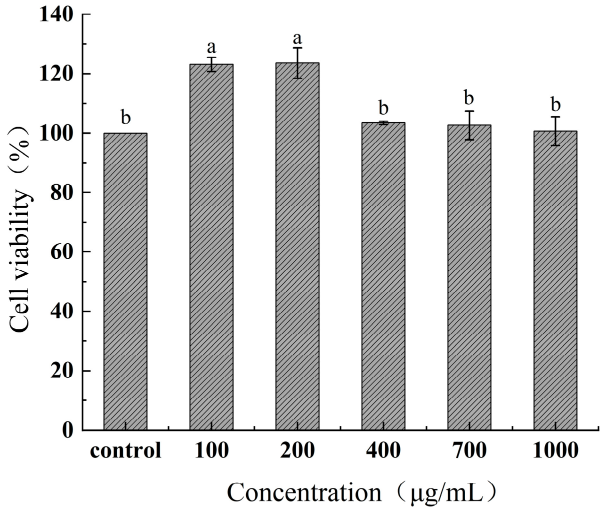

Figure S1. Effect of LBPP-1 on the viability of RAW264.7 cells.
